# Ultrasound-Promoted One-Pot, Three-Component Synthesis of Spiro[indoline-3,1'-pyrazolo[1,2-*b*]phthalazine] Derivatives

**DOI:** 10.3390/molecules17078674

**Published:** 2012-07-23

**Authors:** Juxian Wang, Xiaoguang Bai, Changliang Xu, Yucheng Wang, Wei Lin, Yi Zou, Daqing Shi

**Affiliations:** 1 Institute of Medicinal Biotechnology, Chinese Academy of Medical Sciences and Peking Union, Medical College, Beijing 100050, China; 2 Key Laboratory of Organic Synthesis of Jiangsu Province, College of Chemistry, Chemical Engineering and Materials Science, Soochow University, Suzhou 215123, China

**Keywords:** multicomponent, ultrasound, spiro[indoline-3,1'-pyrazolo[1,2-*b*]phthalazine], spirooxindole

## Abstract

A series of 3'-aminospiro[indoline-3,1'-pyrazolo[1,2-*b*]phthalazine]-2,5',10'-trione derivatives have been synthesized by a one-pot three-component reaction of isatin, malononitrile or ethyl cyanoacetate and phthalhydrazide catalyzed by piperidine under ultrasound irradiation. For comparison the reactions were carried out under both conventional and ultrasonic conditions. In general, improvement in rates and yields were observed when the reactions were carried out under sonication compared with classical conditions.

## 1. Introduction

The challenge of achieving the ideal synthesis has been pursued aggressively since scientists began to construct molecules. Of course, there are many other factors that affect the aspects of synthesis, such as cost, starting material availability, safety, environmental concerns, and overall ease of the process, including work up and purification [1]. Traditional structure-activity relationship evaluations in medicinal chemistry typically involve the preparation of an advanced intermediate that can be analogued readily to introduce the molecular diversity necessary to prepare a collection, or library, of structurally related compounds. One strategy that potentially meets the goals of total synthesis and library production is multicomponent reaction (MCR) chemistry, in which three or more starting materials are brought together in a highly convergent approach to rapidly build up molecular structure and complexity [2,3,4,5,6,7]. According to this method, the products are formed in a single step and the diversity can be achieved simply by varying the reacting components. 

The indole moiety, which is a well-known nitrogen-containing heterocycle, is a common and important feature of a variety of natural products and medicinal agents [8]. Furthermore, it has been reported that the sharing of the indole 3-carbon atom in the formation of spiroindoline derivatives highly enhances the biological activity [9,10,11]. The spirooxindole system is the core structure of many pharmacological agents and natural alkaloids [12,13,14,15,16]. Heterocycles containing bridgehead hydrazine have been studied for over a century due to their pharmacological properties and clinical applications [17,18]. Thus, more and more research briefs have been reported in the past five decades [19,20,21,22,23]. Teimouri [24] reported an one-pot three-component condensation reaction of alkyl isocyanides with dialkyl acetylenedicarboxylates in presence of phthalhydrazide to synthesize dialkyl 3-(alkylamino)-5,10-dioxo-5,10-dihydro-1*H*-pyrazolo[1,2-*b*]phthalazine-1,2-dicarboxylate derivatives. Perumal and Shanthi [25], with their interest in the synthesis of spiroindoline derivatives, have reported the synthesis of pyrazolophthalazinyl spirooxindoles via one-pot three-component reaction catalyzed by L-proline. Recently, Zhang and their partners reported the synthesis of pyrazolophthalazinyl spirooxindoles catalyzed by nickel chloride [26]. However, some of these methods possess some weaknesses such as more expensive catalyst (L-proline), longer reaction time and higher reaction temperature.

In recent years, the application of ultrasound irradiation in organic reactions has been rapidly increasing [27,28,29,30,31]. A large number of organic reactions can be carried out in a higher yield, shorter reaction time and milder conditions under sonication. On the other hand, ultrasonic reactions have been increasingly used as clean, green and environmentally benign routes for the preparation of organic compounds of synthetic and biological values, which is considered to be an important tool for green chemistry in terms of waste minimization and energy conservation [32,33,34,35,36,37,38,39]. Nevertheless, the use of ultrasound in heterocyclic system is not fully explored [40,41,42].

**Scheme 1:**
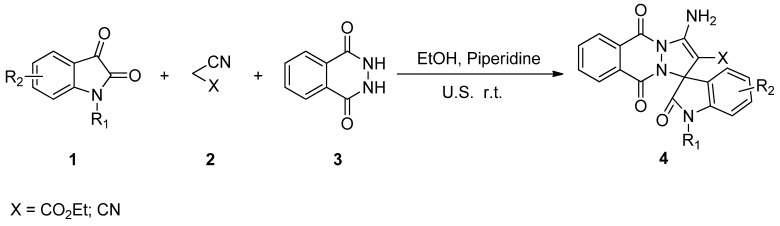
Thesynthesis of 3'-aminospiro[indoline-3,1'-pyrazolo[1,2-*b*]phthalazine]-2,5',10'-trione.

In order to expand the application of ultrasound in the synthesis of heterocyclic compound, herein we report on a facile one-pot, three-component synthesis of 3'-aminospiro[indoline-3,1'-pyrazolo[1,2-*b*]phthalazine]-2,5',10'-trione under ultrasound irradiation (Scheme 1).

## 2. Results and Discussion

To optimize the reaction conditions, the reaction of isatin (**1b**), malononitrile (**2**), and phthalhydrazide (**3**) was selected as the model reaction for the synthesis of spiro[indoline-3,1'-pyrazolo[1,2-*b*]phthalazine] derivatives. Initially, in order to search for a better solvent, the ultrasonic-assisted model reaction was examined using different solvents such as ethanol, methanol, water, acetonitrile and THF, respectively. The results are summarized in Table 1. As can be seen, the reaction could be efficiently carried out in all the tested solvents, although the reaction using ethanol (Table 1, entry 2) as the solvent resulted in higher yields and shorter reaction time than those using methanol, water and acetonitrile as solvent. Thus, ethanol, which additionally is a bio-renewable product of low cost and low toxicity to human health and is relatively non-hazardous to the environment, was chosen as the solvent for all further reactions.

**Table 1:** Optimization of solvent effect on the model reaction ^a^.

Entry	Solvent	Time (h)	Yield ^b^ (%)
1	Methanol	1.5	84
2	Ethanol	0.5	91
3	Water	2	68
4	Acetonitrile	2	65
5	THF	3	70

^a^ *Reaction conditions*: isatin (1 mmol), malononitrile (1 mmol), phthalhydrazide (0.162 g, 1 mmol) and piperidine (0.20 mmol) in solvent (15 mL) at room temperature and the ultrasonic power 250 W, irradiation frequency 25 kHz; ^b^ Yields of isolated products.

Next, we screened different bases for their ability to catalyze the three-component reaction in the same solvent (ethanol). No reaction was observed in the absence of base. The common base such as C_2_H_5_ONa, NaOH, Na_2_CO_3_ and KOH afforded the desired product, but only in moderate to fair yield (Table 2, entries 1–6). Therefore, piperidine was proved the best catalyst for such three-component reaction.

The effect of the concentration of catalyst on the yield of **4b** in ethanol was then explored. The results showed that when the amount of piperidine was increased, an increase in the yields of **4b** was clearly observed. When the concentration of piperidine (mol %) increased from 10% to 30%, the yield of product **4b** increased rapidly from 73% to 91%. The best concentration of piperidine was 20%. The higher concentration of the catalyst did not remarkably increase the yield (Table 2, entries 9 and 10).

In order to further improve the yield and decrease the reaction time, we tried to increase the reaction temperature under ultrasound irradiation. The effect, however, was not remarkable (Table 3, entries 1–3). Consequently, it was indicated that there was no remarkable temperature effect on this reaction.

We also observed the effect of frequency of ultrasound irradiation on the reaction. When the frequency was 25 kHz, the model reaction gave the desired product **4b** in 91% yield at 25 °C. Using 40 kHz did not change reaction yield a considerable amount (90% in the same time). Experiments performed at constant transmitted power but variable frequencies (25 and 40 kHz) show the same trend (Table 3, Entries 3 and 4). It is shown that there is an optimum frequency for effective synthesis of spiro[indoline-3,1'-pyrazolo[1,2-*b*]phthalazine] derivatives in the frequency of 25 kHz.

**Table 2:** Effect of catalyst type and concentration on model reaction ^a^.

Entry	Catalyst	Concentration (mol %)	Time (h)	Yield ^b^ (%)
1	No cat.	0	>3	0
2	C_2_H_5_ONa	10	3	72
3	NaOH	10	3	67
4	Na_2_CO_3_	10	2	65
5	KOH	10	3	55
6	Piperidine	10	2	73
7	Piperidine	15	1.5	84
8	Piperidine	20	0.5	91
9	Piperidine	25	0.5	90
10	Piperidine	30	0.5	90

^a^ *Reaction conditions*: isatin (1 mmol), malononitrile (1 mmol), phthalhydrazide (0.162 g, 1 mmol) and base in ethanol (15 mL) and the ultrasonic power 250 W, irradiation frequency 25 kHz; ^b^ Yields of isolated products.

**Table 3:** Optimization for the Synthesis of **4b** ^a^.

Entry	Frequency (kHz)	Temperature (°C)	Time (h)	Yield ^b^ (%)
1	25	r.t.	0.5	91
2	25	40	0.5	90
3	25	50	0.5	88
4	40	r.t.	0.5	90

^a^ *Reaction conditions*: isatin (1 mmol), malononitrile (1 mmol), phthalhydrazide (0.162 g, 1 mmol) and piperidine (0.20 mmol) in ethanol (15 mL) and the ultrasonic power 250 W, irradiation frequency 25/40 kHz; ^b^ Yields of isolated products.

With this optimum condition in hand, a series of 3'-aminospiro[indoline-3,1'-pyrazolo[1,2-*b*]phthalazine]-2,5',10'-trione derivatives was synthesized in ethanol in the presence of piperidine under sonication . The results are summarized in Table 4.

The reaction was efficiently completed under ultrasound irradiation. From Table 4, we could find that the isatin bearing either electron-withdrawing or electron-donating groups perform well in this reaction. This may be attributed to the reactivity similarity of diversified substituent isatins activated by ultrasonic irradiation in ethanol. However, when ethyl cyanoacetate was used as one of three substrates, the yields of the products were a little lower than the yields of the products in which malononitrile was used and also took comparatively longer reaction time. The reason may be that the nucleophilicity of ethyl cyanoacetate is lower than that of malononitrile.

**Figure 1:**
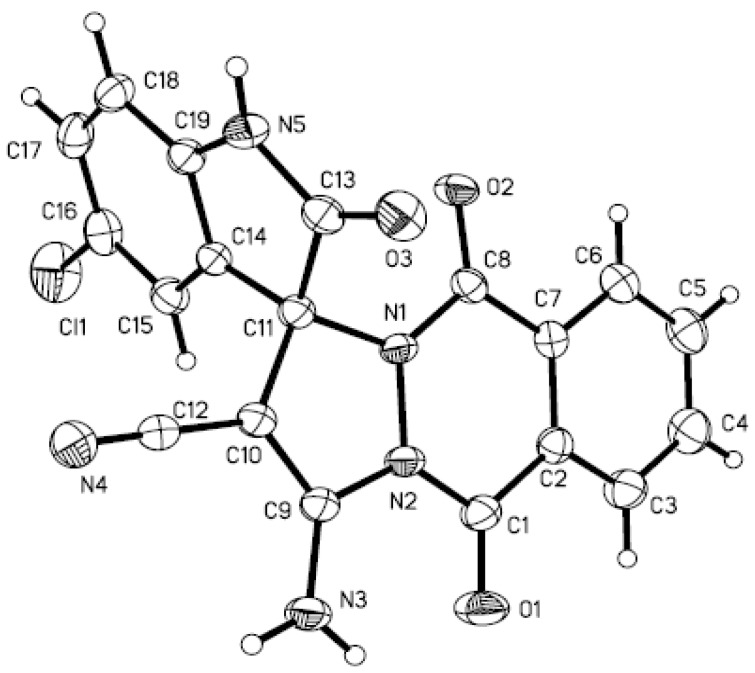
X-ray crystal structure of compound **4d**.

To the best of our knowledge, this new procedure provides the first example of an efficient and ultrasound-promoted approach for the synthesis of spiro[indoline-3,1'-pyrazolo[1,2-*b*]phthalazine] derivatives. This method is the most simple and convenient and would be applicable for the synthesis of different types of nitrogen-containing heterocyclic compounds. The structures of all the synthesized compounds were established by IR, ^1^H-NMR and HRMS. The structure of **4d** was further confirmed by X-ray diffraction analysis. The molecular structure of the product **4d** is shown in Figure 1.

In order to verify the effect of ultrasound irradiation, all the reactions were carried out under the same conditions in absence of ultrasound irradiation (Table 4). The desired products were produced in much longer reaction time (4–9.5 h) and relatively lower yields (42–80%), while under ultrasonic irradiation the products were obtained in 0.5–2 h with the yields of 69–93% (Table 4). The method to obtain the desired products under ultrasonic irradiation offers several significant advantages including faster reaction rates, higher yields and higher purity (without any additional purification step). Thus, ultrasonic irradiation was found to have beneficial effect on the synthesis of spiro[indoline-3,1'-pyrazolo[1,2-*b*]phthalazine] derivatives.

**Table 4:** High efficiency synthesis of spiro[indoline-3,1'-pyrazolo[1,2-*b*]phthalazine] derivatives ^a^.

Entry	Product	X	R_1_	R_2_	Method A ^b^		Method B ^c^	
					Time (h)	Yield ^d^ (%)	Time (h)	Yield ^d^ (%)
1	**4a**	CN	H	5-CH_3_	4	80	1	90
2	**4b**	CN	H	H	4	76	0.5	91
3	**4c**	CN	H	5-Br	4	80	1	92
4	**4d**	CN	H	5-Cl	5	72	1	77
5	**4e**	CN	H	5-F	4	80	0.5	93
6	**4f**	CN	CH_3_	H	4	79	1	93
7	**4g**	CO_2_Et	H	5-CH_3_	8	70	1	82
8	**4h**	CO_2_Et	H	H	8.5	81	1.5	90
9	**4i**	CO_2_Et	H	5-Br	8.5	60	1.5	73
10	**4j**	CO_2_Et	H	5-Cl	7	61	1	80
11	**4k**	CO_2_Et	H	5-F	7.5	69	1	76
12	**4l**	CO_2_Et	CH_3_	H	8	68	1.5	84
13	**4m**	CO_2_Et	H	4-Cl	9.5	42	2	72
14	**4n**	CO_2_Et	H	6-Br	9	58	1.5	69

^a^ *Reaction conditions*: isatin (1 mmol), malononitrile (1 mmol), phthalhydrazide (0.162 g, 1 mmol) and piperidine (0.20 mmol) in ethanol (15 mL) and the ultrasonic power 250 W, irradiation frequency 25 kHz; ^b^ Reaction in ethanol at reflux under high stirring condition; ^c^ Reaction in ethanol at amibent condition under ultrasound irradiation; ^d^ Yields of isolated products.

Through the experiments mentioned above, it was observed that there was a great amount of solid in this system originally because of the bad solubility of isatins and phthalhydrazide in ethanol at room temperature. As the reaction went on under sonication, nevertheless, the reactants were only partially dissolved in the reaction solvent. After 0.5 h it was observed a change in the color of the solid suspended in the reaction mixture thus indicating most probably that reaction occurred. After filtration and analysis such as ^1^H-NMR, it was surprising to find that the product is the desired one. Therefore, in this solid-liquid heterogeneous systems, ultrasound was found to have beneficial effect on the synthesis of spiro[indoline-3,1'-pyrazolo[1,2-*b*]phthalazine] derivatives.

Localized “hot spots” generated from a violent collapse of the bubbles creates a transient high temperature and pressures, inducing molecular fragmentation, and highly reactive species are locally produced. From the Table 4, it is indicated that the obvious difference in the reaction efficiencies with or without sonication suggests again that the reaction under ultrasound condition proceeded in not the same, but in more efficient way than did the reaction under the heating conditions. The yield of the related reaction to synthesize spiro[indoline-3,1'-pyrazolo[1,2-*b*] phthalazine] derivatives is up to 93% under ultrasound condition at the temperature below 30 °C, whereas the yield of the reaction without sonication is only 80% at reflux temperature 80 °C, and the reaction time under sonication is reduced from 4 h to 0.5 h. The implosion of cavities reportedly established an unusual environment for reactions. The gases and vapors inside the cavity are compressed, generating intense heat that raises the temperature of the liquid immediately surrounding the cavity and creates a local hot spot to accelerate the reaction [43,44,45]. In summary, we observed the significant decrease in the reaction time in comparison with conventional methods by our experiments in present system.

**Scheme 2:**
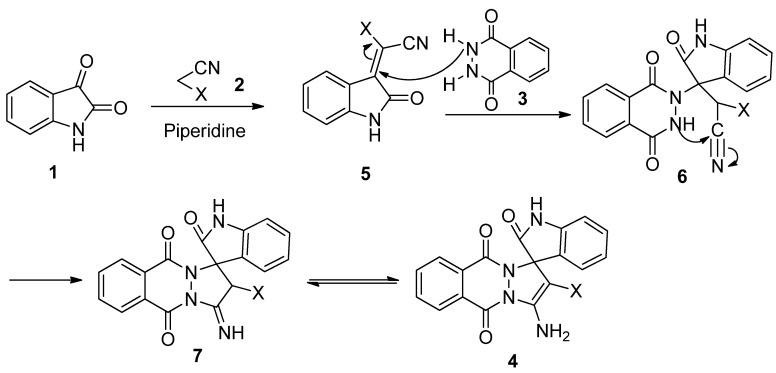
Proposed mechanism for the synthesis of spirooxindole derivatives **4**.

According to the literature [25,26], we proposed the plausible mechanism for the formation of spirooxindole derivatives **4** (Scheme 2). Firstly, we assume that the initial step is a Knoevenagel condensation between isatin **1** and malononitrile or ethyl cyanoacetate **2** catalyzed by piperidine, resulting the intermediate **5**, which suffers a Michael addition of phthalhydrazide **3** to the C=C bond of **5**, followed by cyclization and isomerization to afford the target product **4**.

## 3. Experimental

### 3.1. General

All reagents were purchased from commercial sources and used without further purification. Melting points are uncorrected. IR spectra were recorded on a Varian F-1000 spectrometer in KBr with absorptions in cm^−1^. ^1^H-NMR spectra were determined on a Varian Invoa-300/400 MHz spectrometer in DMSO-*d*_6_ solution. *J* values are in Hz. Chemical shifts are expressed in ppm downfield from internal standard TMS. HRMS data were obtained using Bruker micrOTOF-Q instrument. Sonication was performed in a SY5200DH-T ultrasound cleaner with a frequency of 25 and 40 kHz through manual adjustment and an output power of 250 W. The reaction flask was located at the maximum energy area in the cleaner, and the surface of the reactants was placed slightly lower than the level of the water. Observation of the surface of the reaction solution during vertical adjustment of vessel depth will show the optimum position by the point at which maximum surface disturbance occurs. The addition or removal of water controlled the temperature of the water bath. The temperature of the water bath was controlled at 25–30 °C.

### 3.2. General Procedure under Conventional Conditions (Method A)

A 100 mL flask was charged with the isatin (**1**, 1 mmol), malononitrile or ethyl cyanoacetate (**2**, 1 mmol), phthalhydrazide (**3**, 0.162 g, 1 mmol) and piperidine (0.20 mmol) in ethanol (15 mL). The mixture was stirred at reflux. After the completion of the reaction (monitored by TLC), the solid that precipitated after cooling was collected by filtration. When necessary, the compounds were recrystallized from hot ethanol and DMF to afford pure products.

### 3.3. General Procedure Under Sonochemical Conditions (Method B)

A 100 mL flask was charged with the isatin (**1**, 1 mmol), malononitrile or ethyl cyanoacetate (**2**, 1 mmol), phthalhydrazide (**3**, 0.162 g, 1 mmol) and piperidine (0.20 mmol) in ethanol (15 mL). The mixture was sonicated in the water bath of an ultrasonic cleaner under atmospheric conditions at room temperature. After the completion of the reaction (monitored by TLC), the resulting precipitate was filtered and washed with ethanol to afford the pure product as solid in good to excellent yields.

*3'-Amino-5-methyl-2,5',10'-trioxo-5',10'-dihydrospiro[indoline-3,1'-pyrazolo[1,2-b]phthalazine]-2'-carbonitrile* (**4a**). Yellow solid; m.p. 260–261 °C; IR (KBr, cm^−1^): 3338 (N-H), 3259 (N-H), 3192 (N-H), 2196 (CN), 1744 (C=O), 1658 (C=O), 1566 (C=C), 1497 (ArC=C); ^1^H-NMR (300 MHz, DMSO-*d*_6_): δ (ppm) 2.20 (s, 3H, CH_3_), 6.79 (d, *J* = 7.8 Hz, 1H, ArH), 7.09 (d, *J* = 7.8 Hz, 1H, ArH), 7.30 (s, 1H, ArH), 7.98–8.33 (m, 6H, ArH and NH_2_), 10.81 (s, 1H, NH); HRMS calculated for C_20_H_13_N_5_O_3_Na [M+Na]: 394.0911, found: 394.0912.

*3'-Amino-2,5',10'-trioxo-5',10'-dihydrospiro[indoline-3,1'-pyrazolo[1,2-b]phthalazine]-2'-carbonitrile* (**4b**). Yellow solid; m.p. 263–265 °C (lit. [25] m.p. 269–270 °C); IR (KBr, cm^−1^): 3350 (N-H), 3302 (N-H), 3194 (N-H), 2208 (CN), 1755 (C=O), 1655 (C=O), 1570 (C=C), 1472 (ArC=C); ^1^H-NMR (300 MHz, DMSO-*d*_6_): δ (ppm) 6.91 (d, *J* = 7.8 Hz, 1H, ArH), 6.99 (t, *J* = 7.5 Hz, 1H, ArH), 7.29 (t, *J* = 7.8 Hz, 1H, ArH), 7.46 (d, *J* = 7.2 Hz, 1H, ArH), 7.97–8.34 (m, 6H, ArH and NH_2_), 10.92 (s, 1H, NH); HRMS calculated for C_19_H_11_N_5_O_3_Na [M+Na]: 380.0754, found: 380.0753.

*3'-Amino-5-bromo-2,5',10'-trioxo-5',10'-dihydrospiro[indoline-3,1'-pyrazolo[1,2-b]phthalazine]-2'-carbonitrile* (**4c**). Yellow solid; m.p. 276–277 °C; IR (KBr, cm^−1^): 3406 (N-H), 3309 (N-H), 2970 (C-H Aliph), 2195 (CN), 1745 (C=O), 1655 (C=O), 1566 (C=C), 1475 (ArC=C); ^1^H-NMR (300 MHz, DMSO-*d*_6_): δ (ppm) 6.88 (d, *J* = 5.4 Hz, 1H, ArH), 7.48 (d, *J* = 4.8 Hz, 1H, ArH), 7.81–8.39 (m, 6H, ArH and NH_2_), 11.08 (s, 1H, NH); HRMS calculated for C_19_H_10_^79^BrN_5_O_3_Na [M+Na]: 457.9859, found: 457.9861.

*3'-Amino-5-chloro-2,5',10'-trioxo-5',10'-dihydrospiro[indoline-3,1'-pyrazolo[1,2-b]phthalazine]-2'-carbonitrile* (**4d**). Yellow solid; m.p. 254–255 °C; IR (KBr, cm^−1^): 3354 (N-H), 3250 (N-H), 3193 (N-H), 2206 (CN), 1762 (C=O), 1657 (C=O), 1565 (C=C), 1476 (ArC=C); ^1^H-NMR (400 MHz, DMSO-*d*_6_): δ (ppm) 6.88 (d, *J* = 8.4 Hz, 1H, ArH), 7.31 (d, *J* = 8.4 Hz, 1H, ArH), 7.86 (s, 1H, ArH), 7.96–8.35 (m, 6H, ArH and NH_2_), 11.03 (s, 1H, NH); HRMS calculated for C_19_H_11_^35^ClN_5_O_3_ [M+H]: 392.0544, found: 392.0542.

*3'-Amino-5-fluoro-2,5',10'-trioxo-5',10'-dihydrospiro[indoline-3,1'-pyrazolo[1,2-b]phthalazine]-2'-carbonitrile* (**4e**). Yellow solid; m.p. 258–259 °C; IR (KBr, cm^−1^): 3352 (N-H), 3246 (N-H), 3192 (N-H), 2208 (CN), 1759 (C=O), 1656 (C=O), 1568 (C=C), 1485 (ArC=C); ^1^H-NMR (400 MHz, DMSO-*d*_6_): δ (ppm) 6.93 (q, *J* = 4.0 Hz, 1H, ArH), 7.15 (t, *J* = 8.8 Hz, 1H, ArH), 7.50 (d, *J* = 7.6 Hz, 1H, ArH), 8.01–8.37 (m, 6H, ArH and NH_2_), 10.94 (s, 1H, NH); HRMS calculated for C_19_H_10_FN_5_O_3_Na [M+Na]: 398.0660, found: 398.0660.

*3'-Amino-1-methyl-2,5',10'-trioxo-5',10'-dihydrospiro[indoline-3,1'-pyrazolo[1,2-b]phthalazine]-2'-carbonitrile* (**4f**). White solid; m.p. 287–289 °C (lit. [25] m.p. 282–284 °C); IR (KBr, cm^−1^): 3453 (N-H), 3325 (N-H), 3083 (C-H Ar), 2196 (CN), 1727 (C=O), 1658 (C=O), 1535 (C=C), 1471 (ArC=C); ^1^H-NMR (400 MHz, DMSO-*d*_6_): δ (ppm) 3.25 (s, 3H, CH_3_), 7.10–7.15 (m, 2H, ArH), 7.42 (s, 1H, ArH), 7.54 (s, 1H, ArH), 8.01–8.40 (m, 6H, ArH and NH_2_); HRMS calculated for C_20_H_13_N_5_O_3_ [M]: 371.1016, found: 371.1018.

*Ethyl 3'-amino-5-methyl-2,5',10'-trioxo-5',10'-dihydrospiro[indoline-3,1'-pyrazolo[1,2-b]phthalazine]-2'-carboxylate* (**4g**). Light yellow solid; m.p. 278–280 °C; IR (KBr, cm^−1^): 3439 (N-H), 3333 (N-H), 2982 (C-H Aliph), 1743 (C=O), 1663 (C=O), 1530 (C=C); ^1^H-NMR (400 MHz, DMSO-*d*_6_): δ (ppm) 0.90 (t, *J* = 6.8 Hz, 3H, CH_3_), 2.17 (s, 3H, CH_3_), 3.87 (q, *J* = 6.8 Hz, 2H, CH_2_O), 6.72 (d, *J* = 7.6 Hz, 1H, ArH), 7.02 (d, *J* = 8.0 Hz, 1H, ArH), 7.14 (s, 1H, ArH), 7.99–8.32 (m, 6H, ArH and NH_2_), 10.62 (s, 1H, NH); HRMS calculated for C_22_H_18_N_4_O_5_Na [M+Na]: 441.1169, found: 441.1167.

*Ethyl 3'-amino-2,5',10'-trioxo-5',10'-dihydrospiro[indoline-3,1'-pyrazolo[1,2-b]phthalazine]-2'-carboxylate* (**4h**). Light yellow solid; m.p. 284–285 °C (lit. [25] m.p. 284–286 °C); IR (KBr, cm^−1^): 3440 (N-H), 3332 (N-H), 2984 (C-H Aliph), 1745 (C=O), 1704 (C=O), 1659 (C=O); ^1^H-NMR (300 MHz, DMSO-*d*_6_): δ (ppm) 0.85 (t, *J* = 6.9 Hz, 3H, CH_3_), 3.84 (q, *J* = 6.9 Hz, 2H, CH_2_O), 6.80–6.89 (m, 2H, ArH), 7.20 (t, *J* = 7.5 Hz, 1H, ArH), 7.29 (d, *J* = 7.2 Hz, ArH), 7.97–8.30 (m, 6H, ArH and NH_2_), 10.74 (s, 1H, NH); HRMS calculated for C_21_H_16_N_4_O_5_Na [M+Na]: 427.1013, found: 427.1012.

*Ethyl 3'-amino-5-bromo-2,5',10'-trioxo-5',10'-dihydrospiro[indoline-3,1'-pyrazolo[1,2-b]phthalazine]-2'-carboxylate* (**4i**). Grey solid; m.p. 279–281 °C; IR (KBr, cm^−1^): 3438 (N-H), 3331 (N-H), 2984 (C-H Aliph), 1747 (C=O), 1703 (C=O), 1658 (C=O), 1530 (C=C), 1478 (ArC=C); ^1^H-NMR (400 MHz, DMSO-*d*_6_): δ (ppm) 0.92 (t, *J* = 6.8 Hz, 3H, CH_3_), 3.89 (q, *J* = 6.8 Hz, 2H, CH_2_O), 6.80 (d, *J* = 8.0 Hz, 1H, ArH), 7.40 (d, *J* = 7.6 Hz, 1H, ArH), 7.63 (s, 1H, ArH), 8.01–8.32 (m, 6H, ArH and NH_2_), 10.91 (s, 1H, NH); HRMS calculated for C_21_H_15_^79^BrN_4_O_5_Na [M+Na]: 505.0118, found: 505.0115.

*Ethyl 3'-amino-5-chloro-2,5',10'-trioxo-5',10'-dihydrospiro[indoline-3,1'-pyrazolo[1,2-b]phthalazine]-2'-carboxylate* (**4j**). Green solid; m.p. 284–286 °C; IR (KBr, cm^−1^): 3442 (N-H), 3332 (N-H), 2983 (C-H Aliph), 1750 (C=O), 1705 (C=O), 1660 (C=O), 1530 (C=C), 1480 (ArC=C); ^1^H-NMR (400 MHz, DMSO-*d*_6_): δ (ppm) 0.92 (t, *J* = 6.0 Hz, 3H, CH_3_), 3.89 (q, *J* = 6.8 Hz, 2H, CH_2_O), 6.84 (d, *J* = 8.4 Hz, 1H, ArH), 7.27 (dd, *J_1_* = 2.0 Hz, *J_2_* = 8.0 Hz, 1H, ArH), 7.52 (s, 1H, ArH), 7.99–8.32 (m, 6H, ArH and NH_2_), 10.90 (s, 1H, NH); HRMS calculated for C_21_H_15_^35^ClN_4_O_5_Na [M+Na]: 461.0623, found: 461.0609.

*Ethyl 3'-amino-5-fluoro-2,5',10'-trioxo-5',10'-dihydrospiro[indoline-3,1'-pyrazolo[1,2-b]phthalazine]-2'-carboxylate* (**4k**). White solid; m.p. 294–295 °C; IR (KBr, cm^−1^): 3442 (N-H), 3338 (N-H), 3071 (C-H Ar), 2983 (C-H Aliph), 1749 (C=O), 1706 (C=O), 1656 (C=O), 1530 (C=C), 1488 (ArC=C); ^1^H-NMR (400 MHz, DMSO-*d*_6_): δ (ppm) 0.91 (t, *J* = 6.8 Hz, 3H, CH_3_), 3.88 (q, *J* = 6.8 Hz, 2H, CH_2_O), 6.82 (q, *J* = 4.0 Hz, 1H, ArH), 7.02–7.07 (m, 1H, ArH), 7.33 (dd, *J_1_* = 2.4 Hz, *J_2_* = 8.0 Hz, 1H, ArH), 7.99–8.32 (m, 9H, ArH and NH_2_), 10.79 (s, 1H, NH); HRMS calculated for C_21_H_15_FN_4_O_5_Na [M+Na]: 445.0919, found: 445.0916.

*Ethyl 3'-amino-1-methyl-2,5',10'-trioxo-5',10'-dihydrospiro[indoline-3,1'-pyrazolo[1,2-b]phthalazine]-2'-carboxylate* (**4l**). Light yellow solid; m.p. 288–289 °C; IR (KBr, cm^−1^): 3439 (N-H), 3318 (N-H), 3064 (C-H Ar), 2967 (C-H Aliph), 1729 (C=O), 1705 (C=O), 1668 (C=O), 1616 (ArC=C), 1532 (C=C); ^1^H-NMR (400 MHz, DMSO-*d*_6_): δ (ppm) 0.83 (t, *J* = 7.6 Hz, 3H, CH_3_), 3.22 (s, 3H, CH_3_), 3.81 (q, *J* = 7.6 Hz, 2H, CH_2_O), 6.98 (t, *J* = 7.2 Hz, 1H, ArH), 7.05 (d, *J* = 7.6 Hz, 1H, ArH), 7.31–7.39 (m, 2H, ArH), 8.00–8.32 (m, 6H, ArH and NH_2_); HRMS calculated for C_22_H_18_N_4_O_5_Na [M+Na]: 441.1169, found: 441.1171.

*Ethyl 3'-amino-4-chloro-2,5',10'-trioxo-5',10'-dihydrospiro[indoline-3,1'-pyrazolo[1,2-b]phthalazine]-2'-carboxylate* (**4m**). White solid; m.p. 292–293 °C; IR (KBr, cm^−1^): 3412 (N-H), 3341 (N-H), 2985 (C-H Aliph), 1753 (C=O), 1704 (C=O), 1665 (C=O), 1616 (ArC=C), 1513 (C=C); ^1^H-NMR (400 MHz, DMSO-*d*_6_): δ (ppm) 0.93 (t, *J* = 8.0 Hz, 3H, CH_3_), 3.91 (q, *J* = 8.0 Hz, 2H, CH_2_O), 6.86 (d, *J* = 7.6 Hz, 1H, ArH), 6.92 (d, *J* = 8.4 Hz, 1H, ArH), 7.28 (t, *J* = 8.0 Hz, 1H, ArH), 8.03–8.35 (m, 6H, ArH and NH_2_), 11.02 (s, 1H, NH); HRMS calculated for C_21_H_15_^35^ClN_4_O_5_Na [M+Na]: 461.0623, found: 461.0625.

*Ethyl 3'-amino-6-bromo-2,5',10'-trioxo-5',10'-dihydrospiro[indoline-3,1'-pyrazolo[1,2-b]phthalazine]-2'-carboxylate* (**4n**). Light yellow solid; m.p. 286–287 °C; IR (KBr, cm^−1^): 3447 (N-H), 3341 (N-H), 3258 (N-H), 2985 (C-H Aliph), 1751 (C=O), 1705 (C=O), 1659 (C=O), 1527 (C=C); ^1^H-NMR (400 MHz, DMSO-*d*_6_): δ (ppm) 0.92 (t, *J* = 7.6 Hz, 3H, CH_3_), 3.89 (q, *J* = 7.6 Hz, 2H, CH_2_O), 6.89 (s, 1H, ArH), 7.09 (d, *J* = 7.6 Hz, 1H, ArH), 7.31 (d, *J* = 8.0 Hz, 1H, ArH), 7.99–8.31 (m, 6H, ArH and NH_2_), 10.90 (s, 1H, NH); HRMS calculated for C_21_H_15_^79^BrN_4_O_5_Na [M+Na]: 505.0118, found: 505.0118.

### 3.3. X-ray Crystallography [46]

Yellow crystals of compound **4d** were obtained by slow evaporation from ethanol and DMF. X-ray diffractions were recorded on a Siemens P4 diffractometer. The ORTEP view of the compound with atomic numbering is shown in Figure 1. The structure was solved by using SHELXS97. The structure refinement and data reduction was carried out with SHELXL97 program(s). 

## 4. Conclusions

In summary, we have successfully combined the advantages of ultrasound technology with multicomponent reactions to facilitate the rapid construction of 3'-aminospiro[indoline-3,1'-pyrazolo[1,2-*b*]phthalazine]-2,5',10'-trione derivatives by an one-pot and three-component reaction in ethanol. In comparison with conventional and reported methods, the main advantage of ultrasound application is the significant decrease in the reaction time and all the proposed reactions allowed the preparation of products in good yield without any further complicated refinement.
